# Production of Bioactive Recombinant Bovine Chymosin in Tobacco Plants

**DOI:** 10.3390/ijms17050624

**Published:** 2016-04-28

**Authors:** Zheng-Yi Wei, Yu-Ying Zhang, Yun-Peng Wang, Ming-Xia Fan, Xiao-Fang Zhong, Nuo Xu, Feng Lin, Shao-Chen Xing

**Affiliations:** 1 Agronomy College, Shenyang Agricultural University, No. 120 Dongling Rd., Shenhe Distirct, Shenyang 110866, China; weizy@cjaas.com (Z.-Y.W.); fan_mingxia@foxmail.com (M.-X.F.); 2 Laboratory of Plant Bioreactor and Genetics Engineering, Jilin Provincial Key Laboratory of Agricultural Biotechnology, Agro-Biotechnology Research Institute, Jilin Academy of Agricultural Sciences, No. 1363 Shengtai St., Changchun 130033, China; yuying0609@126.com (Y.-Y.Z.); wangypbio@163.com (Y.-P.W.); zhongxf@cjaas.com (X.-F.Z.); 3 State Key Labortory of Agrobiotechnology, China Agricultural University, No. 2 West Yuanmingyuan Rd., Beijing 100094, China; 4 Chashan Higher Education Zone, Wenzhou University, Wenzhou 325035, China; seer.sino@hotmail.com

**Keywords:** chymosin, rennin, tobacco, milk coagulation, transformation

## Abstract

Chymosin (also known as rennin) plays an essential role in the coagulation of milk in the cheese industry. Chymosin is traditionally extracted from the rumen of calves and is of high cost. Here, we present an alternative method to producing bovine chymosin from transgenic tobacco plants. The *CYM* gene, which encodes a preprochymosin from bovine, was introduced into the tobacco nuclear genome under control of the viral 35S cauliflower mosaic promoter. The integration and transcription of the foreign gene were confirmed with Southern blotting and reverse transcription PCR (RT-PCR) analyses, respectively. Immunoblotting analyses were performed to demonstrate expression of chymosin, and the expression level was quantified by enzyme-linked immunosorbent assay (ELISA). The results indicated recombinant bovine chymosin was successfully expressed at an average level of 83.5 ng/g fresh weight, which is 0.52% of the total soluble protein. The tobacco-derived chymosin exhibited similar native milk coagulation bioactivity as the commercial product extracted from bovine rumen.

## 1. Introduction

Chymosin, more commonly known as rennin, is a key industrial enzyme used to produce cheese, a historically important food in Western countries and one that is in increasing demand in Eastern countries such as China. Cheeses are distinguished by their flavors and/or textures which depend both on their composition and the chymosin used in cheese processing to coagulate the milk [1,2,3,4]. An enzyme with a high clotting to proteolytic activity ratio (C/P value) is preferred because a high proteolytic activity will lead to poorer flavor and/or texture. As bovine chymosin has a high C/P value, it is extremely suitable for cheese production [5]. This is the main reason why bovine chymosin is still widely used and often added to other sources of chymosin such as microorganisms, plants, and other animals [6,7,8,9,10,11,12,13].

Given that chymosin production from calf rumen is limited, alternative methods are needed to produce sufficient bovine chymosin to meet the increasing global demand. The cDNA of the gene encoding bovine chymosin was cloned and analyzed in the early 1980s [14,15], and, since then, many efforts had been made to express recombinant chymosin in various types of heterologous organisms. Successful expression has been achieved in many species such as *Escherichia coli* [16], *Bacillus* species [17], *Aspergillus* species [18,19,20,21], and yeast [22,23,24,25]. Animal-coded chymosins now constitute more than 70% of the global chymosin market [26] with 80% of fermentation chymosin produced as recombinant proteins in microorganisms [27], with protein produced in *Aspergillus* and yeast the most widely used.

Numerous types of proteins including vaccines, antibodies, therapeutic proteins, and industrial enzymes have been successfully expressed using plant expression systems both in intact plants and in plant cell culture [28,29]. Compared with microorganisms, expression of foreign proteins in plants has a number of advantages including superior safety and scale-up capacity, better product quality, more accurate protein folding and post-translational modification. Most importantly, expression in plants offers a low-cost solution for production and storage [30,31,32]. Plant expression systems can be broadly divided into two categories: stable and transient; with stable expression achieved by either the transformation of the nucleus or the plastid, depending on the situation.

Rather surprisingly, given its importance as an industrial enzyme and its potential as a therapeutic enzyme for some disease treatments [33], there have been limited attempts to express chymosin in plants. Willmitzer *et al.* first reported the expression of recombinant chymosin in tobacco leaves [34]. The second example was reported by van Rooijen *et al.*, who developed a seed expression system to achieve a higher expression level [35].

In this paper, we report the expression of bovine chymosin in transgenic tobacco plants. The expression level varied between the different plants, but the highest yield of chymosin was determined to be 83.5 ng/g fresh weight (or 0.52% of total soluble protein (TSP)). The plant-derived chymosin was still active and capable of clotting milk. This study offers an alternatively approach to producing chymosin for cheese production.

## 2. Results

### 2.1. Vector Construction and Tobacco Transformation

**Figure 1:**
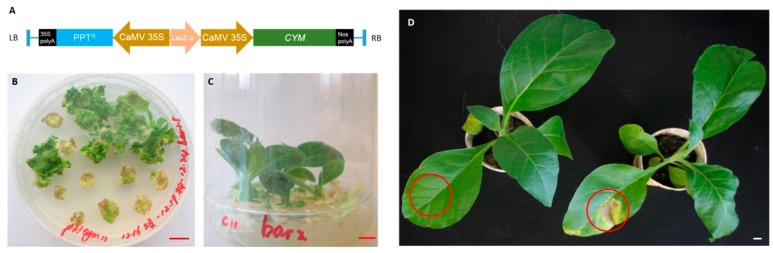
Generation of transgenic plants. (**A**) The T-DNA insert in the bovine preprochymosin (CYM) expression vector p33cym11. LB and RB represent the left and right border of T-DNA region; PPT^R^ represents the glufosinate-resistance encoding gene; CaMV 35S and 35S polyA represent the promoter and the terminator of cauliflower mosaic virus 35S gene respectively; Lac Zα encodes the α-fragment of the enzymy β-galactosidase; Nos polyA represents the terminator of the nopaline synthase gene; (**B**) Resistant shoots regenerated on a selection medium; (**C**) Resistant shoots rooting on rooting medium; (**D**) Plants grown in the greenhouse. After wiping glufosinate solution on the circled portions of the leaves, no necrotic lesions were detected for the transgenic plant (**left**) but obvious symptoms were observed in the wild type (**right**). Scale bars = 1 cm.

The bovine preprochymosin expression vector, p33cym11, was successfully constructed. In Figure 1A, the preprochymosin gene is controlled by the cauliflower mosaic virus 35S promoter (CaMV 35S) and the terminator of nopaline synthase gene (Nos). In the vector, a phosphinothricin acetyltransferase gene, conferring resistance to glufosinate, was used as the selectable marker in tobacco.

Transgenic plants containing the preprochymosin gene were obtained via *Agrobacterium*-mediated leaf disc transformation. The plants were generated on a medium, plus 2 mg/L of glufosinate as a selectable reagent. The transgenic plants displayed glufosinate resistance and showed a normal WT phenotype (Figure 1B–D).

### 2.2. Molecular Analyses

**Figure 2:**
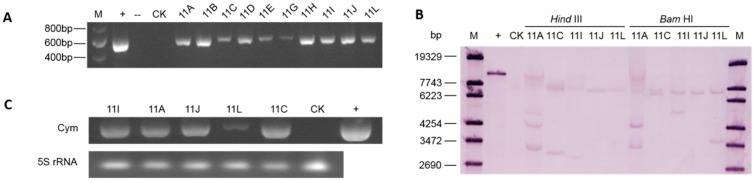
Molecular analysis of transgenic plants. (**A**) PCR analysis of the resistant regenerated plants. M, DNA ladder (Transgene, Beijing, China, cat. No.BM301); +, vector p33cym11 as positive control; --, H_2_O as blank control; CK, wild-type plant; Lane 1-10, resistant plants; (**B**) Southern Blotting analyses using a CYM fragment as probe to hybridize with DNAs digested by *Bam* HI and *Hind* III. M, DNA ladder (lambda DNA digested by *Sty* I); +, vector p33cym11 as positive control; CK, wild-type plant; 11A, 11C, 11I, 11J, and 11L are different transgenic lines; (**C**) Transcription analysis of the *CYM* gene in transgenic plants. Cym indicates the specific bands for the *CYM* gene, 5S rRNA shows the RNA amount used in the analysis. 11I, 11A, 11J, 11L, and 11C are different transgenic lines; +, vector p33cym11 as positive control; CK, wild-type plant.

Genomic DNA was extracted from glufosinate-resistant plants to perform molecular analyses. PCR analyses, carried out as a preliminary screen to confirm the existence of the preprochymosin gene, *CYM*, revealed that most of the resistant plants were positive (Figure 2A). The integration of the preprochymosin gene was further confirmed with Southern blotting. The results demonstrated that all the plants tested were transgenic but contained different copies of the foreign gene (Figure 2B). There was one copy in line 11J and two or more copies in other four lines. The RT-PCR results indicated that the *CYM* gene was transcribed in all the tested plants (Figure 2C), but there is seemingly no relation between the transcription level and copy number inserted. Line 11L, for instance, had two copies of the target gene, but showed the lowest accumulation of mRNA, while line 11J harboring a single copy was observed with a much higher accumulation.

### 2.3. Expression of Recombinant Bovine Chymosin and Detection of Activity

**Figure 3:**
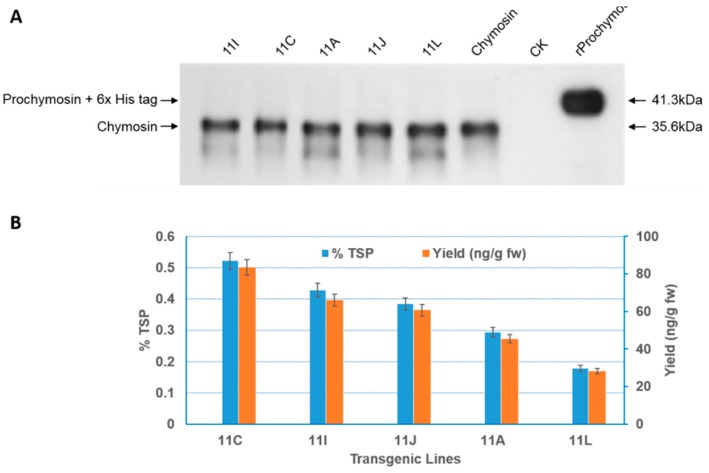
Expression of recombinant bovine chymosin in transgenic tobacco plants. (**A**) Immunoblotting analysis; (**B**) Yield of recombinant bovine chymosin in transgenic plants. Chymosin, CK, and rProchymosin represent commercial chymosin isolated from calf, crude TSP from wild-type plant and recombinant prochymosin with 6× His tag from *E. coli* respectively; 11A, 11C, 11I, 11J, and 11L represent different individual transgenic plant lines or crude TSP from them. Yield data are collected from three independent duplicates for each line.

Immunoblotting confirmed the successful expression of bovine chymosin in TSP extracted from the plants (Figure 3A). A quantitative analysis by ELISA indicated that expression levels varied from 18.1 to 83.5 ng/g fresh weight, meaning 0.18% to 0.52% of TSP (Figure 3B).

**Figure 4:**
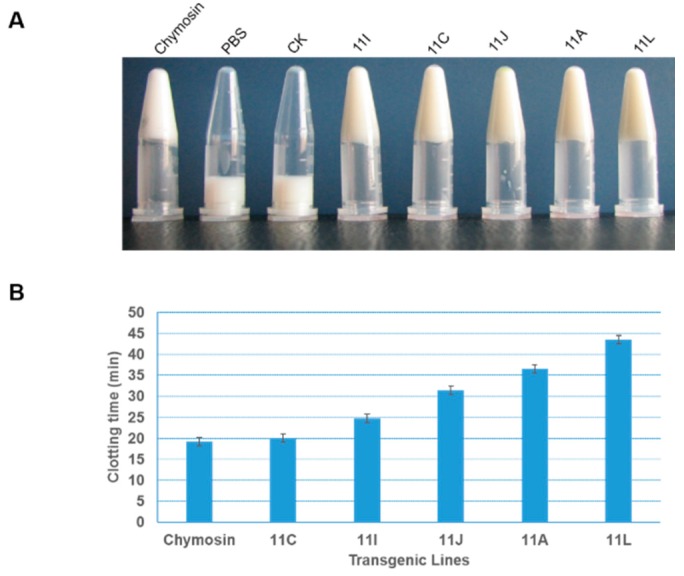
Milk clotting tests for recombinant bovine chymosin. The status of milk clotting (**A**) and the time consumed (**B**) are shown. Chymosin, PBS, and CK represent the commercial chymosin from calf, PBS buffer, and crude TSP from wild-type plant respectively; 11A, 11C, 11I, 11J, and 11L represent crude TSPs from the transgenic plants accordingly. Data of clotting time are collected from three independent repeats.

A bioactivity assay was performed to test whether the plant-derived chymosin was still functional. In this assay, the time taken for fresh milk to be clotted by the plant TSP was assessed. All plants expressing chymosin displayed natural bioactivity. As the expression level varied in different transgenic plants, the crude chymosin exhibited different levels of bioactivity, at a milk-clotting time ranging from 20.5 to 46.3 min (Figure 4). Interestingly, the accumulation of mRNA possibly was correlated with the yield of protein.

## 3. Discussion

Although bovine chymosin has been expressed in many microorganisms, production still cannot meet market demands [36]. Expression of chymosin in plants might help address this shortfall since the GM plant offers an additional source of supply and its production can potentially scale up at low cast. Our report here offers an example of this expression platform serving the cheese production industry. We show here that active bovine chymosin can be expressed in tobacco plants with a yield of 83.5 mg/kg fresh weight (approximately 0.52% TSP). This level is similar to the previous report by Willmitzer *et al.* [34], who also expressed chymosin in tobacco leaves, but is much lower than that reported in flex seeds and oilseed rape by van Rooijen *et al.* [35].

One way to improve upon the expression levels of chymosin reported here would be to use a codon-optimized gene, as genes from different species may have a particular genetic codon usage bias [37], and this may crucially impact on the accumulation of heterogenic proteins in plant. Codon-optimized genes according to the codon usage bias of target host species [38] guarantee the translation efficiency. Van Rooijen *et al.* [35] claimed that the optimized bovine prochymosin gene is suitable to expression in at least 22 plant species listed in the patent, but they only showed the details in flax and oilseed rape. Indeed, this helped to increase the percentage of chymosin in the total seed protein of flax (up to around 2.6% TPS) and oilseed rape (average of 4.43% TPS). In the present report, the DNA sequence of the bovine preprochymosin is taken from a calf without optimization, which could be one of the reasons leading to a lower expression level; thus, codon optimizing should be added to further studies.

In addition, there are potentials to modify the transcription of the target gene to improve the expression of recombinant proteins in plants [39], and the tissue-specific promoters would be an example by means of modulating the transcription of preprochymosin gene [34,35]. One report did not seem to have a higher accumulation of chymosin in leaves (only up to 0.5% TSP) [34], while another report worked well in seeds [35]. In our case, a constitute promoter CaMV 35S was used to ensure the expression in all tissues, as we expected to harvest the whole plant for protein extraction. It might be better to use an inducible, non-tissue-specific promoter, as it may reduce the adverse effects on plant growth.

Stability of foreign proteins expressed in plants could also be a critical issue for their accumulation. Multiple options could be adopted as solutions [40,41,42]. Secretory expression or the sorting of proteins to organelles have been applied to partition heterologous proteins from cytoplasm, where various house-keeping proteases are present, and hence to reduce the degradation risk of the target proteins. Numerous studies have demonstrated that heterologous proteins are well protected in apoplast [43,44,45]. We retained the secretory peptide of the prochymosin in this study with the expectation to have the protein secreted to the intercellular space. On the other hand, whether sorting chymosin to various organelles could be advantageous over intercellular space should be investigated; therefore, constructs having chymosin ORF fused with organelle-specific transit peptides should be tested in the future.

Chymosin self-activates at pH between 4 and 5 [46], but the pH of plant cytoplasm is slightly alkaline [47]. In order to obtain active chymosin, the enzyme should be stored in a location of lower pH value. Vacuole and intercellular spaces are ideal choices [48]. According to our results, the chymosin extracted from tobacco leaves exhibited milk-clotting activity, which implies that the enzyme had been activated in the plant tissue, as the pH value of crude extract and milk are approximately 7.0 and 6.5 respectively, which is outside the range of 4.0 to 5.0 for self-activation, providing indirect evidence that the recombinant chymosin has been correctly transited to the intercellular space accordingly. Moreover, the presence of active chymosin added to the significance of this work: The crude extract could be directly applied to milk for cheese processing without protein purification, and this might reduce the production cost.

## 4. Materials and Methods

### 4.1. Vector Construction

The expression vector harboring the preprochymosin gene from bovine (*Bostaurus domesticus*) is a derivative of the binary transformation vector pCAMBIA3301 (the glufosinate resistant version of the binary vector pCAMBIA-1301, accession No. AF234297) and was constructed by replacing the GUS gene by the bovine preprochymosin gene. Basically, primers cymF (5′-TATAGATCTTCGACCTCGAGATGAGGTGTCTCGTGGTGCTACTTGC-3′) and cymR (5′-GGAGGCCTGGATCGACTAGTGGTGGTGGTGGTGGTGGATGGCTTTG-3′) were used to amplify the bovine preprochymosin gene using plasmid pGEM-T-CYM (kindly provided by Professor Zhen-Nai Yang) as a template. The product was subsequently inserted into the backbone of pCAMBIA3301 digested by *Nco* I and *Bst* EII (New England Biolabs, Beijing, China) to create the final expression vector, namely p33cym11, by the in-fusion method [49]. The vector was verified by sequencing and then transferred to the *Agrobacterium tumefaciens* strain EHA105 via a freeze/thaw method [50].

### 4.2. Tobacco Transformation and Generation of Transgenic Tobacco

Tobacco (*Nicotiana tabacum cv.* Petite Havana SRI) seeds were surface sterilized and sown on Murashige and Skoog (MS) medium [51] in the culture room at 25 °C with a 16 h/8 h light-dark photoperiod. The sterile leaves were collected as explants for transformation after one month. The vector was transferred into tobacco leaf discs via *Agrobacterium*-mediated transformation. After co-culturing for 3 days, the leaf discs were transferred to rest media (MS medium plus 0.2 mg/L α-naphthylacetic acid, 2 mg/L 6-benzylaminopurine and 500 mg/L carbenicillin) to inhibit the growth of *Agrobacteria* for 7 days and then removed to selection medium (rest media plus 2 mg/L glufosinate) for regeneration. The selection media were renewed every 15 days until the resistant shoots were obtained. The shoots were transferred to a rooting medium (MS medium supplemented with 2 mg/L glufosinate) for rooting. Leaves from resistant plants were used to perform molecular analyses, and the transgenic plants confirmed with Southern hybridization were transferred to soil and cultured in a green house. During the cultivation, the plants were screen again by wiping leaves with 2 mg/L of glufosinate solution, and the uninjured plants were maintained to grow until seeds were collected.

### 4.3. Molecular Analysis

#### 4.3.1. PCR and Southern Blotting Analysis

The genomic DNA were extracted from resistant regenerated plants and used as templates to carry out PCR reaction to confirm the existence of the preprochymosin gene in the tobacco genome; DNA from the wild-type plant served as a negative control. The primers were cymdF (5′-CGTGCCCCTGACCAACTACCTG-3′) and cymdR (5′-CGGAGGGGGTCAGTGGGTACAT-3′). The PCR-positive plants were further analyzed via Southern blotting: 20 μg of genomic DNA was digested by *Bam* HI and *Hind* III, respectively, and applied to a 0.8% (*w*/*v*) agarose gel for electrophoresing. The electrophoresis was run for 8 h to separate fragments at a current of 5 V/cm in the TAE buffer. DNAs were then transferred to a positive-charged Hybond-N^+^ nylon membrane (Roche, Shanghai, China) and fixed by UV cross-linking. The DNA-fixed membrane was hybridized at 42 °C for 16 h with the digoxigenin (DIG)-labeled Cym probe in a hybridization oven, and the immune-link detection procedure and visualization was performed according to the manufacturer’s manual for the DIG High Prime DNA Labeling and Detection Starter Kit I (Roche, Shanghai, China).

#### 4.3.2. RT-PCR Analyses

Total RNA was extracted from potential transformants to perform RT-PCR analyses. Two microgram of total RNA from each plant was used as a template for reverse transcription reactions to synthesize cDNA, with the RNA from wild-type plant used as a negative control. The cDNA were used as template to perform PCR analyses with the primer cymdF and cymdR, under the same condition as the PCR analyses. The endogenous 5S rRNA was chosen to quantify the initial RNA amount.

### 4.4. Immunoblotting and ELISA Analyses

Mature leaves of T_1_ plants were collected to extract total soluble protein (TSP). Approximately 200 mg of leaf tissue was ground into a fine powder in liquid nitrogen, and 400 μL of PBS (pH7.4) buffer was added to the powder and mixed vigorously for 5 min; the TSP was in the supernatant after centrifuging and then quantified by the Bradford method [52]. For each sample, 50 μg of TSP were separated via sodium dodecyl sulfate-polyacrylamide gel electrophoresis (SDS-PAGE) on a 12% separating gel. The blotting was carried out followed the standard protocol [53]. The primary antibody against bovine chymosin was produced by us from rabbit and anti-rabbit secondary antibody, conjugated with horse radish peroxidase purchased from Sino Biological Inc. (Beijing, China). Blots were visualized on an X-ray film by an enhanced chemiluminescence (ECL) method. The same TSP was used to carry out the ELISA analysis with the same primary antibody against bovine chymosin, following a protocol previously described [54]. The TSP from the wild-type plant was used as a negative control for both of the assays above, and the chymosin expressed from *E. coli* as well as commercial bovine chymosin (TCI, Shanghai, China) isolated from calf rumen were used as positive controls for the immunoblotting.

### 4.5. Bioactivity Detection of Recombinant Bovine Chymosin

A milk coagulation assay was used to detect the bioactivity of the recombinant bovine chymosin from tobacco by a method described previously [55]. Briefly, 400 μL of fresh milk, 55 μL of 100 mM CaCl_2_, and 50 μL of crude TSP were added to 1.5-mL tubes and well mixed; then, the tubes were incubated in a shaker at 35 °C at a speed of 70 rpm. Commercial bovine chymosin (TCI, Shanghai, China) isolated from calf rumen (0.1 mg/mL), the TSP from the wild-type plant, and PBS buffer were used as positive, negative, and blank controls, respectively.
